# True Unipolar ECG Machine for Wilson Central Terminal Measurements

**DOI:** 10.1155/2015/586397

**Published:** 2015-10-01

**Authors:** Gaetano D. Gargiulo

**Affiliations:** The MARCS Institute, University of Western Sydney, Kingswood, NSW 2747, Australia

## Abstract

Since its invention (more than 80 years ago), modern electrocardiography has employed a supposedly stable voltage reference (with little variation during the cardiac cycle) for half of the signals. This reference, known by the name of “Wilson Central Terminal” in honor of its inventor, is obtained by averaging the three active limb electrode voltages measured with respect to the return ground electrode. However, concerns have been raised by researchers about problems (biasing and misdiagnosis) associated with the ambiguous value and behavior of this reference voltage, which requires perfect and balanced contact of at least four electrodes to work properly. The Wilson Central Terminal has received scant research attention in the last few decades even though consideration of recent widespread medical practice (limb electrodes are repositioned closer to the torso for resting electrocardiography) has also sparkled concerns about the validity and diagnostic fitness of leads not referred to the Wilson Central Terminal. Using a true unipolar electrocardiography device capable of precisely measuring the Wilson Central Terminal, we show its unpredictable variability during the cardiac cycle and confirm that the integrity of cardinal leads is compromised as well as the Wilson Central Terminal when limb electrodes are placed close to the torso.

## 1. Introduction

Surface electrocardiography, by definition, is the time-domain representation of the electrical activity of the beating heart inside the chest, measured as voltage variation over time by surface electrodes placed in contact with the skin. Surface electrocardiography is represented by a vector quantity ($\overset{-}{P}$) rotating around a fixed point (the electrical center of the heart) in the body frontal plane describing an angle (*α*) with a fixed direction identified by an imaginary line crossing the shoulders [1]. This definition was originally outlined in 1908 by E. Einthoven, later revised in 1931 by F. N. Wilson, who named the fixed point as the “central terminal,” and further modified in 1942 by E. Goldberger, who invented the augmented leads [1]. From 1942, the mentioned definition and associated recording guidelines produced the so-called 12-lead ECG system, which is currently considered to be the best practice [1, 2].

**Figure 1:**
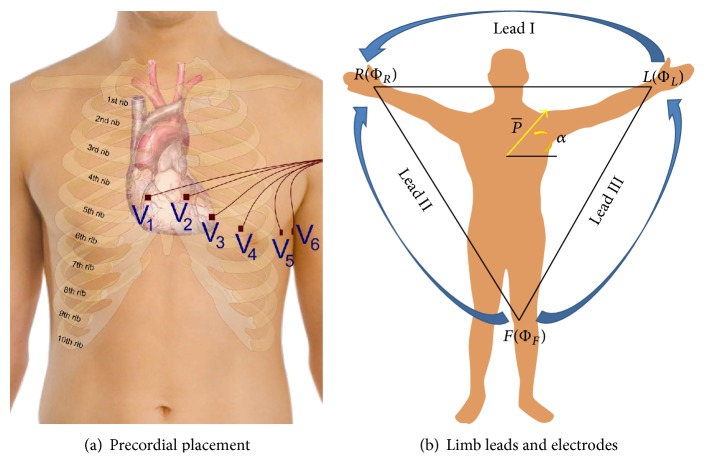
Twelve-lead ECG electrode placement and lead names [1] (a and b).

The 12-lead ECG is so called because it produces twelve ECG signals. It uses a reference electrode placed on the right leg (RL) and nine exploring electrodes: three limb electrodes placed on the right arm (RA), left arm (LA), and left leg (LL) and six electrodes placed over the torso near the heart [1]. Electrode positioning and signals recordable from the six electrodes over the torso have been named precordial leads (precordials) and are also known simply as “chest leads” (see Figure 1(a)) or as *V*_1_ to *V*_6_ leads, while the signals recordable from the limbs have been named cardinal (or fundamental) Einthoven leads (see Figure 1(b)) and are referred to as Lead I, Lead II, and Lead III or simply as “limb leads”:

 Lead I: *V*_I_ = Φ_*L*_ − Φ_*R*_;
 Lead II: *V*_II_ = Φ_*F*_ − Φ_*R*_;
 Lead III: *V*_III_ = Φ_*F*_ − Φ_*L*_;

with

 
*V*_I_ being the voltage of Lead I;
 
*V*_II_ the voltage of Lead II;
 
*V*_III_ the voltage of Lead III;
 Φ_*L*_ the potential at the left arm**∗**;
 Φ_*R*_ the potential at the right arm**∗**;
 Φ_*F*_ the potential at the left leg**∗**
 
**∗**referred to the electrode on the right leg (Φ_RL_).

The augmented leads are measured as the voltage difference between each of the limb potentials and the average of the other two limb potentials. For example, the augmented lead *aV*_*F*_ is measured as

(1) $$\begin{array}{c} {aV}_{F}=\Phi_{F}-\frac{\left(\Phi_{L}+\Phi_{R}\right)}{2}. \end{array}$$

Because all of the limb potentials are implicitly referred to the potential of the right leg, it is possible to infer that cardinal leads are recorded as twice the voltage difference. For example, assuming the potential of the right leg Φ_RL_ being measured with respect to a point at a neutral potential (i.e., earth ground), Lead I can be rewritten as

(2) $$\begin{array}{c} V_{I}=\left(\;\Phi_{L}-\Phi_{RL}\;\right)-\left(\;\Phi_{R}-\Phi_{RL}\;\right). \end{array}$$

Although at first glance it may seem that the potential Φ_RL_ will cancel out; due to the nonideal (not infinite) capacity to reject common signals that are present simultaneously at the inputs, known as the Common Mode Rejection Ratio (CMRR) [3, 4] of the employed amplifiers, any contact imbalance between the three electrodes may cause signal quality degradation and unpredictable drift of slow components. Intuitively, the effect of contact impedance imbalance gets worse when considering augmented leads as they require perfectly balanced contact from all of the four limb electrodes [1, 5, 6]. This is counterintuitive as the circuit that they form in the human body is an equilateral triangle that does not take into account the RL voltage at all (see Figure 1(b)).

Similarly, the voltage of a virtual point called the Wilson Central Terminal (WCT) is subtracted from each of the precordials' electrode potentials. The WCT is obtained by averaging the potential at the limbs referred to the reference electrode on the right leg using three identical resistors (5 kΩ or higher) connected to a single point [1]:

(3) $$\begin{array}{c} \Phi_{WCT}=\frac{\left(\Phi_{L}+\Phi_{R}+\Phi_{F}\right)}{3}. \end{array}$$

Although Wilson himself used to refer to the precordials as “unipolar” [7], this has been repeatedly pointed out as a misnomer due to the repeated voltage difference required to obtain them [8–11]. It has also been demonstrated that the WCT cannot be considered a “null” potential [8, 9] nor should it be confused with the real center of the heart potential, because the ECG signals travel through different trunks of an inhomogeneous volume conductor and can be exposed to different sources of noise such as different expositions to RF fields and artifacts [9, 12]. In 1954, Frank [8] was the first to raise concerns about the potential fluctuations in the WCT during a cardiac cycle and how they could bias the ECG measurement [8, 13, 14]. He predicted that within a few years a new, refined cardiac conduction theory and ECG system able to work without the WCT would emerge. In the early days of modern electrocardiography, other researchers were also able to confirm that the WCT is not constant during the cardiac cycle. Confirmation of errors and variability of the WCT during the cardiac cycle have been measured employing an “integrator electrode.” This procedure requires the entire human body to be encased in a metal structure and then immersed in water (neutral reference) during the measurement of ECG. Unfortunately, due to the cumbersomeness of the measurement process, this technique was used only for few experimental trials [15, 16]. In recent years, the significance of the WCT and even its physical location has also been debated [9, 10, 17]. However, aside from notable attempts in the 1940s and 1950s [14, 18, 19], until our study, the WCT has never been correctly measured without a cumbersome procedure and in a repeatable way.

In this context, one must mention that not only has the WCT received scant research attention in the last few decades, but also there is a generalized lack of modern studies about the general placement of electrodes and the impact that electrode misplacement (particularly when intentional) may have upon diagnosis. Current common widespread medical practice is to move the limb electrodes to positions closer to the torso (shoulders and hips or sides of the navel). This is thought to reduce the obtrusiveness of the ECG recording as cables are not spread all over the body, which is particularly advantageous during stress recordings. However, there is evidence [20] that limb electrode positioning that affects the QRS influences the diagnosis of ischemic (including chronic) heart diseases [21, 22]. Although there is some evidence that in healthy subjects the variation in the ECGs imposed by alteration of the limb electrodes can be classified only as statistically relevant and not as clinically relevant [23], due to the significant shift in cardiac axis and waveform amplitude that can be observed in both ECG planes when the limb electrodes are in positions different from the standard ones [24], standardized recommendation for ECG clinical practice [25] confirms that misplacement of limb electrodes should be avoided [22] or used only where strictly necessary (i.e., stress test) and always noted on the recording [25].

Over the past two years, we have developed a new electrocardiographic device [3, 11, 12, 26–28] that allows real-time visualization and precise measurement of the WCT amplitude, shape, and variations; using this device we show that the WCT exhibits a clinically significant variation (>0.1 mV or >1 mm [2, 14]) across different recordings and during the course of the same recording. For the evaluation presented in this paper we have partially reused the unipolar ECG data that have been recorded from a small population of healthy subjects who volunteered during a previous study [11, 12, 26, 27] and agreed to have the data analyzed for publication purposes by expert cardiologists. The subject population comprises five males covering the age span of 29–36 years with an average age of 32.5 years. None of the subjects had a history of cardiac illness, and all the recordings presented normal sinus rhythms. We also recorded data from one volunteer subject again, performing two recordings consecutively to show the effect of placing the limb electrodes near the torso on cardinal leads.

## 2. Experimental Section

Our principal hypotheses for this study are as follows.

1. The WCT is not a stable voltage reference exhibiting a clinically significant voltage variation.
2. Moving the limb electrodes to a position near the torso can affect the shape and amplitude of cardinal leads as well as the WCT.

To demonstrate our hypotheses, we firstly introduce the true unipolar machine and a measurement technique that allows us to reliably measure and store the WCT; then, we present the data processing with a full example of WCT variability across the cardiac cycle and through a recording. Lastly, we show the effect that the placement of the limb electrodes near the torso (from ankles and wrists to hips, sides of the navel, and shoulders) has on limb leads and the WCT [25].

### 2.1. Hardware Development

**Figure 2:**
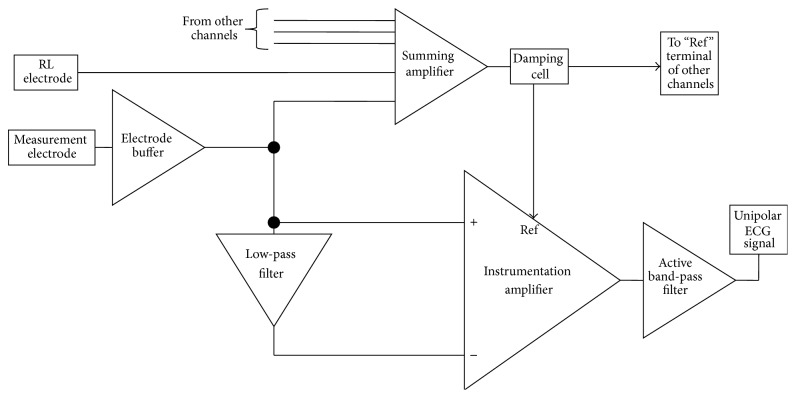
Block diagram of proposed ECG system.

Our hardware front-end and its pilot evaluation are properly described in [11, 12, 26–28]. However, for the sake of completeness, in this section we give a brief summary of the measurement hardware employed in this study. In Figure 2, we show a functional block diagram of the ECG amplifier (one single channel). In principle, we regard the unipolar ECG measurement as a combined observation of noise and useful signal. It is thus possible to measure the local signal of interest by subtracting the local noise (or what is regarded as such) from the measured signal. As it is possible to observe in Figure 2, the measured signal (measurement electrode) is fed to an instrumentation amplifier that subtracts from the signal a low-pass version of the same signal (the low-pass cut-off frequency is set at 0.1 Hz). With this technique, a pseudo-high-pass DC-coupled ECG front-end is achieved, preserving the ultrahigh input of the amplifier, which allows the use of dry electrodes. Experiments confirmed that the low-pass filter used to achieve the pseudo-high-pass filter can be implemented with passive components and its cut-off frequency can be positioned at very low frequency (i.e., 0.01 Hz), employing high value capacitors and resistors. This is possible because the ultrahigh-input impedance of the instrumentation amplifier employed can cope with several MΩ of impedance.

Amplifier referencing is achieved via the reference terminal of the instrumentation amplifier labeled as “Ref.” The Ref terminal receives a damped version (low passed) of the summation of all the electrode signals and of the RL electrode. This technique, which is also known as “modified ground bootstrapping” [3, 12, 29–31], similar to the standard ground bootstrapping [3, 32], achieves power-line noise and electrodic noise suppression without the use of a driven right-leg technique [33, 34].

**Figure 3:**
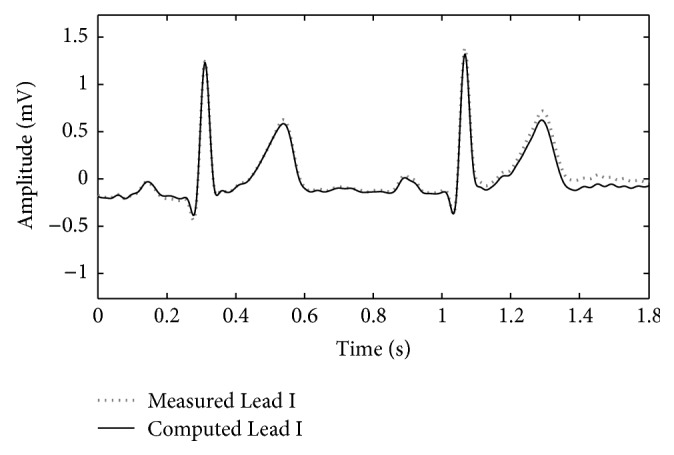
Example of a traditional ECG lead reconstruction from unipolar leads (point-to-point subtraction) (the data used to plot the image were recorded for the study [12]).

Signals recorded using this instrument can be regarded as being referred directly to the right leg. Therefore, a simple point-by-point subtraction between recorded signals allows real-time calculation of the 12-lead ECG. In Figure 3, an example of the calculation for Lead I is shown. In this example, prerecorded left-arm and right-arm signals have been simply subtracted to obtain Lead I. With this recording technique, the WCT is simply calculable from a point-by-point average of the recorded limb potentials. In order to allow reconstruction of traditional precordials (obtained by simple point-by-point subtraction of the WCT), our precordials are also directly referred to the potential of RL [11, 12, 27]. In our previous pilot study [12, 28], we demonstrated that correlation between the reconstructed signals and parallel recording of traditional signals exceeds 90% with minimal differences, which are due to components' tolerance [11, 12, 26].

### 2.2. Measurement

For this study, we calculate the WCT by averaging the prerecorded limb potentials. As we have shown in our previous analysis, the WCT is profoundly different across subjects and may have the shape of ECG leads with sometimes very well marked characteristic waveforms such as a P wave, a QRS complex, and a T wave. For this reason, we measure the WCT's amplitude at its largest feature that is expected to normally coincide with the QRS-like complex. In other words, we measure this amplitude as the peak-to-peak amplitude. In this study, we show that the amplitude of the WCT varies during a recording and that, similar to what has been already demonstrated for standard ECG leads [20], its shape and amplitude are affected by the positions of limb electrodes. Using a case study we have also been able to justify the commonly observed shift of the cardiac axis towards the vertical direction [20, 23, 24].

## 3. Results and Discussion

**Figure 4:**
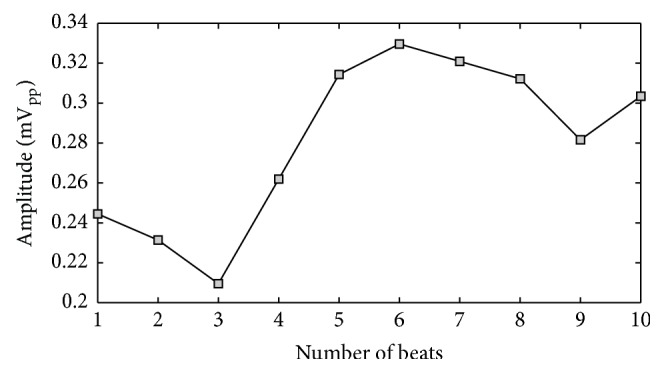
Variation in WCT amplitude measured across 10 consecutive beats selected starting from a random beat within the recording (see text).

**Figure 5:**
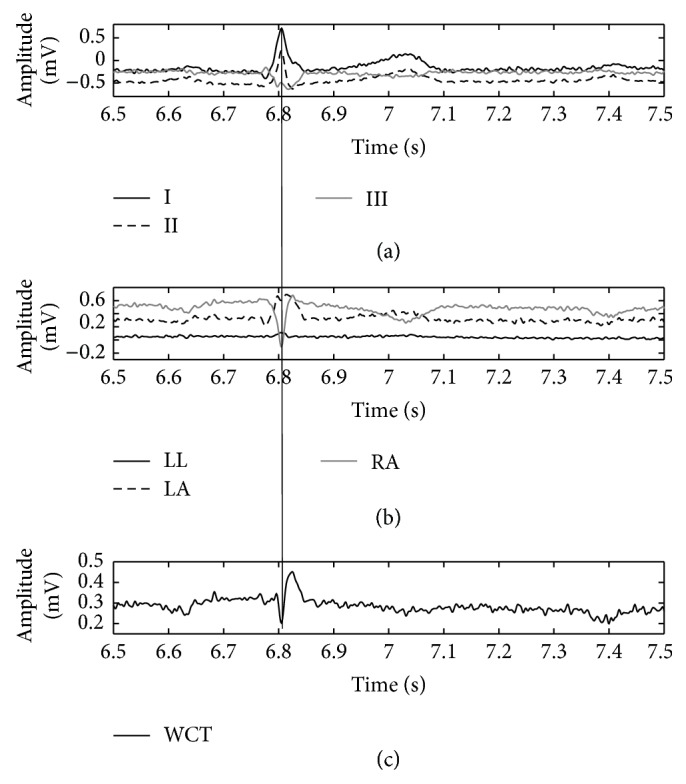
Direct comparison of WCT (c) with cardinal limb leads (a) and true unipolar components (b) when limb electrodes are placed on wrists and ankles. The QRS fiducial point is marked (thin vertical line) using Lead II as the reference.

**Figure 6:**
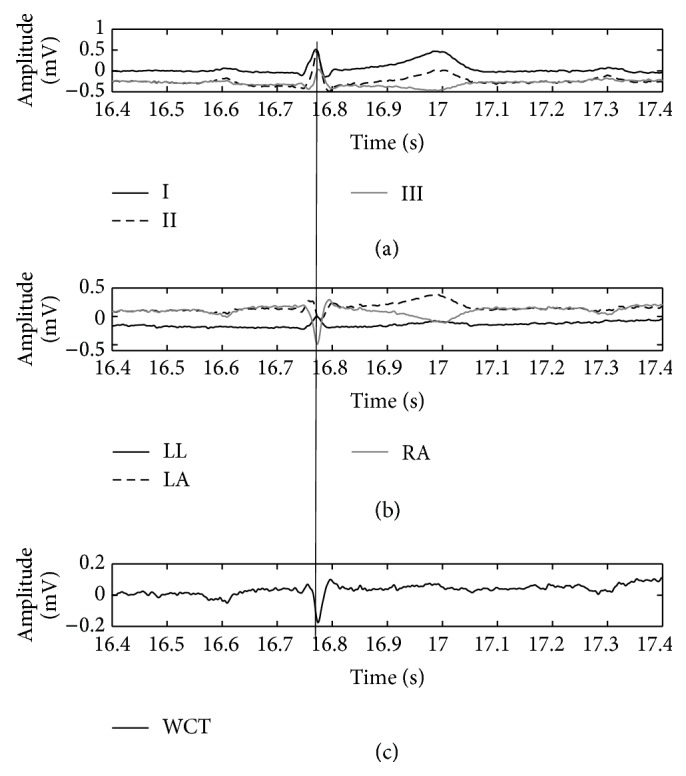
Direct comparison of WCT (c) with cardinal limb leads (a) and true unipolar components (b) when limb electrodes are placed on hips and shoulders. The QRS fiducial point is marked (thin vertical line) using Lead II as the reference.

(1) The WCT exhibits clinically relevant (>0.1 mV or >1 mm) amplitude variability during each cardiac cycle as well as clinically significant variation during the recording. In order to show this variability in a concise way, we selected a random starting point within the recording and measured the amplitude of the WCT for 10 consecutive beats after that point. As it is possible to observe from Figure 4, all of the 10 considered beats have an amplitude larger than 0.1 mV; moreover, between beat #3 and beat #6 there is the largest large extent of variability (0.12 mV) between cardiac cycles.
(2) Similar analyses performed for the other subjects of our database [11, 12, 26, 27] yield similar results.
(3) Our general WCT amplitudes are in accordance with values presented in the literature. We recall that amplitudes for the WCT of the order of 0.2 mV were already measured during a historical experiment that made use of a cumbersome procedure. During the experiment a volunteer was immersed in water whilst being encased in a metal structure called an “integrator electrode” [15, 16, 18, 35]. Our device instead allows continuous WCT precise measurement by recording straight from the limb electrodes.
(4) The WCT noise level is influenced directly by all three limb potentials; hence movement artifacts on any of the limbs or any contact impedance imbalance between the limb electrodes will directly affect the WCT signal quality and possibly degrade the precordials. Because the true unipolar device records limb components, noise affecting one of the limbs can be evaluated beforehand, and hence operators can decide not to use the WCT if it is compromised without experiencing loss of the entire set of precordials. To this extent, the amplitude of the WCT seems to be dominated by the right-arm (RA) component (which is the largest component observable from Figure 5(b)); similar observations were made for the other subjects enrolled in our pilot study and hence we can confirm the previous hypothesis that WCT may impair chest exploration due to biasing imposed by the right arm [14].
(5) The position of the limb electrodes directly affects the shape of the leads and WCT. A simple comparison of Figures 5 and 6 reveals that the QRS feature of the WCT is distorted. When electrodes are moved to the shoulders and hips (see Figure 6), the S-wave decreases in favor of a larger R-wave and this is particularly visible in Lead III, where the QRS is clearly larger.
(6) In unipolar components, there is a marked increase in the amplitude of the LL component and a reversion of the LA component polarity. For these reasons it is possible to say that the increase of information carried by the lower body (LL) and the simultaneous distortion of the information carried by the upper body (LA) justify the deviation of the cardiac axis in favor of more vertical directions, as observed in literature [24]. This finding is supported by an intuitive analysis of the correct formula for the calculation of the cardiac axis. Recalling that the cardiac axis is calculated by [36]
  (4) $$\begin{array}{c} \text{Cardiac Axis}=\pm \tan^{-1}\frac{{aV}_{F}}{I} \end{array}$$
 which can be expressed in unipolar components as [28]
  (5) $$\begin{array}{c} \text{Cardiac Axis}=\pm \tan^{-1}\frac{LL-\left(\left(RA+LA\right)/2\right)}{LA-RA}, \end{array}$$
 it is easy to conclude that a marked increase in LL alone will increase the vertical component of the vector $\overset{-}{P}$ representing the cardiac activity, shifting the value of its angle *α* towards a steeper value; one may note that a reversion of the LA polarity may also contribute to an increase of the numerator of the cardiac axis calculation formula, which, when limb electrodes are moved closer to the torso, is also always accompanied by a reduction of Lead I (the denominator), which may further increase the shift of *α* toward the vertical axis.

Lastly, because the signals recorded with the true unipolar device are linearly independent, similar to what is done with EEG recordings, it is possible to increase the space of signals via rereferencing. Namely, the number of signal traces obtainable from the 10 placed electrodes will increase from twelve to at least thirty (nine independent unipolar ones, nine referred to the common average, and the twelve traditional signals), thereby increasing the redundancy of information present in the ECG, as has been sought since its invention more than 80 years ago [1]. In other words, a corollary of this new method is that the current practice is at the same time improved (more robustness to noise, larger redundancy of information, and visualization of WCT) and preserved (the traditional signal and diagnostic method are also useable). It is notable that reconstruction of 12-lead ECG based upon point-to-point subtraction of components can be more robust to noise. This is because signal analysts (medical practitioners annotating the ECG with or without the aid of automated procedures) will be able to estimate the signal-to-noise ratio of each individual component (such as power-line noise and artifacts) and operate individual differentiated and customized software filters on the components before reconstructing the signal [11, 12, 26, 27].

## 4. Conclusions

We presented experimental evidence that the WCT is not a stable reference for ECG leads through the cardiac cycle, that its shape and amplitude (measured peak to peak) are comparable with the amplitude of other ECG leads, and most importantly that it shows clinically significant amplitude variability during the recording. With this study we also show that the WCT, like the limb leads, is directly affected by alteration of the electrode position and therefore it can pass this additional bias to precordials with unforeseen effects upon diagnosis.

Using our device, in this study, we have also been able to justify the shift of the cardiac axis toward the vertical direction that has been observed in several independent studies when limb electrodes are placed closer to the torso (i.e., stress ECG). Hence, since our analysis and experiment confirm concerns about the alteration of all standard leads when limbs electrodes are placed closer to the torso, we conclude that this practice should be avoided or used only where strictly necessary (i.e., when recording is not possible otherwise).

Lastly, our technique for measurement of ECG signals, allowing calculation of the WCT and standard 12-lead ECG, offers the construction of a larger space of signals, which adds redundancy to the ECG, as has been sought since its invention more than 80 years ago [1]. We are currently seeking ethical clearance for a large trial to confirm the extent and impact of our findings, particularly concerning the effect of the currently widespread practice of placing the limb electrodes closer to the torso.
